# Insufficient Physical Activity and Sedentary Behaviors among Medical Students during the COVID-19 Lockdown: Findings from a Cross-Sectional Study in Pakistan

**DOI:** 10.3390/ijerph181910257

**Published:** 2021-09-29

**Authors:** Irfan Ullah, Md. Saiful Islam, Sajjad Ali, Hashaam Jamil, Muhammad Junaid Tahir, Aatik Arsh, Jaffer Shah, Sheikh Mohammed Shariful Islam

**Affiliations:** 1 Kabir Medical College, Gandhara University, Peshawar 25000, Pakistan; irfanullahecp2@gmail.com; 2 Department of Public Health and Informatics, Jahangirnagar University, Savar, Dhaka 1342, Bangladesh; 3 Centre for Advanced Research Excellence in Public Health, Savar, Dhaka 1342, Bangladesh; 4 Ziauddin Medical College, Ziauddin University, Karachi 75600, Pakistan; sajjad110@live.com; 5 Ameer-ud-Din Medical College, Lahore 54000, Pakistan; hashaamjamil543@gmail.com (H.J.); junaid262626@gmail.com (M.J.T.); 6 Lahore General Hospital, Lahore 54000, Pakistan; 7 Institute of Physical Medicine & Rehabilitation, Hayatabad, Peshawar 25000, Pakistan; aatikarshkmu@yahoo.com; 8 Department of Global Health, Drexel University College of Medicine, Philadelphia, PA 19102, USA; jaffer.shah@drexel.edu; 9 Institute for Physical Activity and Nutrition, School of Exercise and Nutrition Sciences, Faculty of Health, Deakin University, Burwood, VIC 3125, Australia; shariful.islam@deakin.edu.au

**Keywords:** exercise, physical activity, BMI, medical students, COVID-19, Pakistan

## Abstract

*Background:* The unprecedented COVID-19 pandemic has resulted in social distancing and isolation which leads to insufficient physical activity and thereby increases sedentary behaviors. Hence, this study aimed to assess the prevalence of insufficient physical activity and sedentary behaviors among medical students during the COVID-19 lockdown in Pakistan, and to determine their associated factors. *Methods:* A cross-sectional online survey was carried out among 407 medical students from the Punjab and Sindh provinces between May and June 2020. To collect data, an e-questionnaire was sent to obtain informed consent along with questions concerning socio-demographics as well as an International Physical Activity Questionnaires–Short Form (IPAQ–SF). *Results:* As per the IPAQ, almost five in ten participants were physically inactive (48.2%), and 45.2% reported sedentary behaviors. Participants with insufficient physical activity were more likely to report sedentary behaviors than their counterparts (AOR = 2.53; 95% CI = 1.66–3.85, *p* < 0.001). The odds of insufficient physical activity were higher among the participants who did not strictly follow the COVID-19 preventive measures (AOR = 2.51; 95% CI = 1.35–4.69, *p* = 0.004); similarly, there were increased odds of sedentary behaviors observed among participants within a normal weight range compared to those who were underweight (AOR = 2.69; 95% CI = 1.76–4.11, *p* < 0.001). *Conclusions:* Insufficient physical activity and sedentary behavior are prevalent among medical students in Pakistan during the COVID-19 pandemic. These findings indicate the importance of establishing tailored policies and programs to encourage young adults to engage in physical activity.

## 1. Introduction

In early December 2019, several cases of pneumonia, caused by a novel beta-coronavirus (the 2019 novel coronavirus) were identified in Wuhan, the capital city of Hubei, China [1]. The World Health Organization (WHO) declared coronavirus disease 2019 (COVID-19) a global pandemic on 11 March 2020, when the registered cases of COVID-19 reached 118,000, and the number of deaths reached 4291 across 114 countries [2]. On 23 March 2020, registered cases in Pakistan reached 892 with six deaths; in order to prevent the spread of this disease, the government of Pakistan enforced a countrywide lockdown on 24 March 2020, which continued for more than a month [3]. Furthermore, strict Standard Operating Procedures (SOPs) (i.e., wearing masks, keeping six-feet distance, etc.) were implemented to reduce the spread of the disease in the work environment. A countrywide closure of all educational institutes took place from 13 March 2020 [4]; however, online classes were offered by some educational institutions. All these precautionary measures have led to a decrease in physical activity patterns among the student population.

Insufficient physical activity causes adverse changes in health, such as muscular atrophy, bone weakness, obesity, cardiovascular changes, etc. [5]. Many studies have shown the positive effects of physical exercise in improving general health, as well as immune functions [6,7]. From a public health perspective, moderate physical activities have shown a remarkable effect on well-being. According to Martin et al. and Ravalli et al., moderate physical exercise has been linked to the improvement of upper respiratory tract infections caused by COVID-19 [8,9]. Moreover, physical activity has been shown to alleviate symptoms of depression and anxiety and enhance mental well-being [10]. In contrast, there has been some linkage between exercising during acute infection with an increased risk of adverse effects such as myocardial damage [11,12].

Previous studies have suggested that the restrictions placed on public health linked to the COVID-19 pandemic seem to have contributed to an increase in sedentary behaviors and insufficient activity [13,14,15]. A study based on Italian medical students showed decreased levels of total physical activity and an increased frequency of sedentary behaviors during the lockdown [16]. Similar findings have also been discovered among the student population in other jurisdictions including in Canada, Italy, Spain, Switzerland, and so on [17,18,19,20,21]. In addition, a Brazilian study on the behavioral and psychosocial aspects of social exclusion during the COVID-19 pandemic found that compared to people with improved mental health, those with higher levels of stress, depression, and anxiety performed less physical activity [22]. Conversely, some studies noted increased levels of physical activity in some population groups [23]

However, data are scarce on the impact of the COVD-19 pandemic on lifestyle behaviors in medical students, particularly from the South Asian region. Although compared to other students, medical students possess better knowledge surrounding healthy lifestyles and the importance of physical activity [24], there is no evidence to indicate that medical students translate this knowledge into practice. Consequently, the objective of this study was to assess the prevalence of insufficient activity and sedentary behaviors among medical students during the COVID-19 lockdown in Pakistan, and to determine the factors associated with insufficient activity and sedentary behaviors.

## 2. Materials and Methods

### 2.1. Participants and Procedure

A cross-sectional online survey was conducted between May and June 2020, involving medical students of the Punjab and Sindh provinces during the COVID-19 pandemic while the lockdown/stay-home order was strictly imposed in Pakistan. The target population included students who were seeking the Bachelor of Medicine, Bachelor of Surgery (MBBS) degree. After obtaining informed consent along with all study measures in the Google survey form, a shareable link was generated. The link was disseminated throughout social networking sites (e.g., Facebook, WhatsApp, Instagram, etc.) targeting medical students with a view to obtaining a good response during the pandemic. The inclusion criteria of the participants included (i) being medical students, (ii) residing in Pakistan, (iii) having access to a smartphone and social networking sites, and (iv) a willingness to take part in the survey. The participants who did not entirely complete the survey were excluded from the final sample.

### 2.2. Sample Size Estimation 

The sample size required for this cross-sectional analysis was calculated using the Raosoft sample size calculator with a 95% confidence interval, 90 percent as the online survey response rate, and a 5% margin of error. Thus, it was calculated that the minimum number of participants needed for this study was 400. Incomplete submission of the survey questionnaire was not possible due to the feature in Google Forms that prevented the submission of partially answered or partially filled questions, which did not require a five percent or 10 percent increase in the required sample size. 

### 2.3. Ethics

The present study followed the highest level of ethical perspective suggested by the Helsinki Declaration (Revised 2013) and the International Ethical Guidelines for Human Research in Health (2016). The formal ethics approval was taken from the Institute of Physical Medicine and Rehabilitation (IPMR), Peshawar [DIR/IPMR-EB/00388]. The aims and objectives were clearly documented in the informed consent section and all the participants willingly consented to take part in the survey. The data presented herein are anonymous and do not constitute an in-person interaction with human subjects.

### 2.4. Measures

A web-based survey was employed to gather information from the participants. This consisted of questions related to the evaluation of physical activity during the COVID-19 lockdown period, as well as socio-demographics and COVID-19 related measures. 

#### 2.4.1. Socio-Demographic Information

The socio-demographic questions included age, gender, year of academic study, smoking history (yes or no), family system (nuclear or joint), medical university category (public or private), and height and weight. BMI was calculated as the weight in kilograms divided by the height in meters square, kg/m^2^, and was classified into three classes according to the Asian-Pacific cutoff points: underweight (<18.5 kg/m^2^), normal/lean (18.5–24.9 kg/m^2^), or overweight/obese (>25 kg/m^2^) [25,26,27,28].

#### 2.4.2. COVID-19 Related Questions

The four questions related to COVID-19 that were asked were: (i) Do you have any COVID-19 related symptoms? (ii) Have you been diagnosed with COVID-19 by a health professional? (iii) Have you been living with a person diagnosed with COVID-19, and (iv) Are you following the COVID-19 Standard Operating Procedures (SOPs)?

#### 2.4.3. International Physical Activity Questionnaires–Short Form (IPAQ–SF)

In the present study, the IPAQ–SF was used to assess physical activity and sedentary behaviors [29]. The IPAQ–SF is a seven-item scale which based on the frequency and duration of activities, estimates the level of physical activity over the last seven days with good reliability and validity [30,31]. IPAQ–SF is also validated in the Pakistani population, in the Urdu language [32]. Physical activity is measured on the basis of the energy expenditure through vigorous activities, moderate activities, walking, and sitting time. The energy expenditure is measured by metabolic equivalents multiplied by time and week, such as vigorous MET (vigorous minutes × days × 8.0), moderate MET (moderate minutes × days × 4.0), and walking MET (walking minutes × days × 3.3). Total physical activity is measured by summation of the vigorous MET, moderate MET, and walking MET. Total Physical activity is graded as a low activity (<600 MET per week), moderate activity (between 600–3000 MET per week), and high activity (>3000 MET per week) [33]. In our study, the 600 MET cutoff was used to determine those who were physically inactive (<600 MET per week) and physically active (≥600 MET per week). Sedentary behaviors for each participant were categorized by those who reported ≥8 h/day sedentary activities, defined as “*those activities in a sitting, reclining, or lying position (except sleep) requiring very low energy expenditure*”, and examples included sitting/lying down, reading, watching TV, computer use, playing video games, etc., as used in previous studies [34,35,36].

### 2.5. Statistical Analysis

Data were analyzed using the statistical software Statistical Package for Social Sciences (SPSS) version 25. Some basic statistical analyses including means, standard deviations, frequencies, percentages, etc., were computed. Bivariate and multiple logistic regression analyses were performed to determine the significant associations between insufficient activity and sedentary behaviors. The outcome variables including physically inactive and sedentary behaviors for each participant were categorized by those who had <600 MET per week and ≥8 h/day sedentary activities, respectively. A *p*-value less than 0.05 was considered to be statistically significant. 

## 3. Results

### 3.1. General Profile of Participants

An overview of the frequencies of the demographic variables of the final sample (n = 407) is presented in Table 1. A total of 407 participants with a mean age of 21.88 (SD = 1.56) were included in the final analysis. Of them, the majority were female medical students (54.8%), from public medical universities (64.1%), studying in their final year (28.5%), from urban areas (86.0%), and living with nuclear families (76.4%). Regarding the participants’ BMI, 66.1% had a normal weight, while a minority were underweight (13.8%) or overweight/obese (20.1%). Most reported themselves as non-smokers (97.3%).

Only 6.1% participants reported that they had COVID-19-like symptoms. 1.7% reported that they had been diagnosed with COVID-19, and 4.4% were living together with COVID-19-infected persons. In addition, most reported that they followed the COVID-19 standard operating procedures (82.8%). 

### 3.2. Physical Activity

As per the IPAQ, almost five in ten participants were physically inactive (48.2%); in contrast, 42.8% were moderately active, and only 9.1% were highly active. Using bivariate regression analysis, age, study year, BMI, living with COVID-19 infected persons, following the SOPs, and sedentary behaviors, were selected (*p* < 0.25) as candidates of multivariable regression analysis (Table 1). The participants who did not strictly follow the SOPs were 2.5 times more likely to be physically inactive than those who did (AOR = 2.51; 95% CI = 1.35–4.69, *p* = 0.004). Moreover, the participants reporting sedentary behaviors had greater odds of being physically inactive than those who did not report sedentary behaviors (AOR = 2.69; 95% CI = 1.76–4.11, *p* < 0.001). 

### 3.3. Sedentary Behaviors

As per the IPAQ, a sizeable majority of the participants reported sedentary behaviors (45.2%). Using bivariate regression analysis, medical university, study year, residence, BMI, COVID-19-like symptoms, COVID-19 diagnosis, living with COVID-19-infected persons, and insufficient activity, were selected (*p* < 0.25) as candidates of multivariable regression analysis (Table 2). Participants within the normal weight range had increased odds compared to those who were underweight (AOR = 2.18; 95% CI = 1.14–4.19, *p* = 0.019). Participants reporting insufficient activity were 2.5 times more likely to report sedentary behaviors than their more active counterparts (AOR = 2.53; 95% CI = 1.66–3.85, *p* < 0.001).

## 4. Discussion

The current study presents a clear picture of the effects of the COVID-19 pandemic and the countrywide lockdown on the lifestyles of the medical students. To our knowledge, this is the only study conducted in Pakistan that investigated insufficient activity patterns and sedentary behaviors among medical students during the pandemic. We found that 48.2 percent of medical students were physically inactive during the COVID-19 pandemic. Surprisingly, a study which took place prior to the COVID-19 pandemic reported that 49% of medical students were physically inactive [37]. This clearly shows that the countrywide lockdown, restricted social interaction, and lack of access to public venues resulted in no significant change to the physical activity of medical students. There remains the question of why physical activity has stayed consistent, and whether the impacts on physical activity habits would have been the same if the lockdown had lasted longer. Students’ sedentary behavior patterns are affected by their living environment [38], and it appears that for medical students, their environment might not support physical activity. Another factor to consider in the case of medical students is that their training in encouraging healthy behaviors may have affected their choice to exercise at home [19].

According to a study by Hong et al. [39], the cognitive constructs of health consciousness and self-efficacy were found to have a significant impact on an individual’s attitude toward physical exercise, behavioral intention, and behavior. Health consciousness has a stronger influence on attitudes toward physical exercise, but self-efficacy has a stronger influence on behavioral intention and action. This can be specifically compared to our study, where we found that medical students who did not strictly follow the SOPs were significantly more likely to be physically inactive (*p* = 0.004). Despite knowing the severity of the disease and the consequences it has on the healthcare system and those surrounding it, some of the medical students seemed to show careless attitudes towards their own health by not participating in physical activities and not following the SOPs accordingly. 

Through our analysis, we have seen that a sizeable majority of medical students (45.6%) reported sedentary behaviors. In the present study, medical students who were physically inactive were 2.5 times more likely to engage in sedentary behaviors compared to physically active students. Sedentary behaviors over long periods of time cause adaptations that alter and degrade the fitness level mitochondrial function, and are thus linked to disease aggravation [40,41]. It is likely that limited physical exercise for long periods of time during the COVID-19 emergency will lead to sedentary tendencies [36].

High sedentary habits were also associated with BMI, where students with a normal weight had increased odds compared to those who were underweight (*p* = 0.019). This implies that social restrictions had no significant impact on students with a low BMI, in terms of their sedentary behavior. Students with a low BMI remained consistent in their physical activity and utilized their time in activities which decreased their sedentary behaviors. In contrast, people with a normal weight adopt sedentary habits more since they believe themselves to be much healthier than their underweight or overweight counterparts. However, in contrast to a study conducted on university students in Italy during lockdown, sitting time increased in students with a normal or underweight BMI [19]. 

The significance of physical exercise and limiting sedentary habits cannot be emphasized enough, given their positive impact on overall health as well as particular aspects connected to the COVID-19 pandemic, such as immune system regulation [42]. To maintain a physically active lifestyle throughout the pandemic, the WHO and many other professional associations encourage the adoption of particular exercise programs and daily tactics, including home-based exercise programs [43,44].

### Limitations

The present study has several drawbacks, which should be disclosed. Firstly, the cross-sectional study recruited relatively small samples using the convenient sampling technique, and only included students from the Sindh and Punjab provinces. Therefore, the results cannot be generalized to the whole medical student body of Pakistan. A future study involving a larger sample size is warranted in this respect. Secondly, our results do not show the physical activity level among the students before the lockdown was enforced across the whole country. Therefore, it is challenging to evaluate the outcomes of the study and to draw a clear inference on the physical activities of medical students amid lockdown. Thirdly, the lockdown was imposed in Pakistan on 13 March 2020, and we collected the data two months later; our sample population could have adapted to the lockdown situation and may have applied healthy activities such as exercise, which could make our results appear to indicate more physical activity than was initially the case. Lastly, because all of the data were self-reported, it is likely that individuals either underestimated or exaggerated their weight and the amount of time they spent in sedentary behavior or physical activity.

## 5. Conclusions

Insufficient physical activity and sedentary behavior are present in a substantial majority of medical students in Pakistan during the COVID-19 pandemic. The significant associated factors for insufficient physical activity and sedentary behavior were BMI, COVID-19 related symptoms, exposure to COVID-19 patients, and following the SOPs. The study suggests that medical students, perhaps knowing the importance of physical activity with regard to their health, have continued to maintain or even increased their exercise levels and patterns during the current lockdown situation. Online resources for maintaining an exercise routine (e.g., the use of online Zumba classes) to get the maximum benefit of home-based exercise are recommended. The government of Pakistan should encourage students who lack the opportunity to avail online exercising programs and sports club memberships. There is a need to encourage physical activity among medical students to fight COVID-19. This is necessary since medical students are the physicians of tomorrow, and therefore should set an example for their future patients as well.

## Figures and Tables

**Table 1:** Distribution of all variables, and regression analysis predicting insufficient activity.

Variables	Overall N = 407	Insufficient Activity					
		No	Yes	Bivariate Analysis		Multivariable Analysis	
		n (%)	n (%)	COR (95% CI)	*p*-Value	AOR (95% CI)	*p*-Value
Gender							
Female	223 (54.8)	111 (49.8)	112 (50.2)	1.201 (0.812–1.776)	0.358	—	—
Male	184 (45.2)	100 (54.3)	84 (45.7)	Ref.			
Age							
≤20 years	82 (20.1)	35 (42.7)	47 (57.3)	1.442 (0.729–2.855)	0.293	1.456 (0.535–3.96)	0.462
21–23 years	269 (66.1)	147 (54.6)	122 (45.4)	0.891 (0.501–1.587)	0.696	0.809 (0.403–1.622)	0.55
≥24 years	56 (13.8)	29 (51.8)	27 (48.2)	Ref.		Ref.	
Medical university							
Public	261 (64.1)	131 (50.2)	130 (49.8)	1.203 (0.801–1.806)	0.373	—	—
Private	146 (35.9)	80 (54.8)	66 (45.2)	Ref.			
Year of study							
2nd	53 (13)	23 (43.4)	30 (56.6)	1.826 (0.688–4.849)	0.227	2.251 (0.798–6.348)	0.125
3rd	105 (25.8)	52 (49.5)	53 (50.5)	1.427 (0.582–3.5)	0.437	2.315 (0.808–6.629)	0.118
4th	109 (26.8)	58 (53.2)	51 (46.8)	1.231 (0.503–3.011)	0.649	2.453 (0.811–7.415)	0.112
5th (Final)	116 (28.5)	64 (55.2)	52 (44.8)	1.137 (0.467–2.77)	0.777	1.636 (0.518–5.168)	0.402
1st	24 (5.9)	14 (58.3)	10 (41.7)	Ref.		Ref.	
Residence							
Urban	350 (86.0)	179 (51.1)	171 (48.9)	1.194 (0.46–3.097)	0.715	—	—
Semi-urban	39 (9.6)	22 (56.4)	17 (43.6)	0.966 (0.314–2.974)	0.952	—	—
Rural	18 (4.4)	10 (55.6)	8 (44.4)	Ref.			
Family type							
Joint	96 (23.6)	48 (50)	48 (50)	1.101 (0.697–1.741)	0.679	—	—
Nuclear	311 (76.4)	163 (52.4)	148 (47.6)	Ref.			
BMI							
Underweight	56 (13.8)	25 (44.6)	31 (55.4)	1.45 (0.813–2.587)	0.208	1.648 (0.891–3.051)	0.112
Overweight/obese	82 (20.1)	41 (50)	41 (50)	1.169 (0.713–1.918)	0.535	1.267 (0.732–2.194)	0.398
Normal	269 (66.1)	145 (53.9)	124 (46.1)	Ref.		Ref.	
Cigarette smoking							
Yes	11 (2.7)	4 (36.4)	7 (63.6)	1.917 (0.552–6.651)	0.305	—	—
No	396 (97.3)	207 (52.3)	189 (47.7)	Ref.			
Do you have any COVID-19 related symptoms?							
Yes	25 (6.1)	11 (44)	14 (56)	1.626 (0.597–4.43)	0.342	—	—
No	341 (83.8)	177 (51.9)	164 (48.1)	1.184 (0.617–2.273)	0.612	—	—
May be	41 (10.1)	23 (56.1)	18 (43.9)	Ref.			
Have you been diagnosed with COVID-19 by a health professional?							
No	400 (98.3)	207 (51.7)	193 (48.3)	1.243 (0.275–5.626)	0.778	—	—
Yes	7 (1.7)	4 (57.1)	3 (42.9)	Ref.			
Have you been living with a person diagnosed with COVID-19?							
Yes	18 (4.4)	6 (33.3)	12 (66.7)	2.228 (0.82–6.057)	0.116	1.855 (0.634–5.423)	0.259
No	389 (95.6)	205 (52.7)	184 (47.3)	Ref.		Ref.	
Are you following COVID-19 Standard Operating Procedures (SOPs)?							
No	15 (3.7)	9 (60)	6 (40)	0.783 (0.273–2.248)	0.649	0.799 (0.257–2.482)	0.698
Not sure	55 (13.5)	20 (36.4)	35 (63.6)	2.055 (1.139–3.706)	0.017	2.514 (1.349–4.687)	0.004
Yes	337 (82.8)	182 (54)	155 (46)	Ref.		Ref.	
Insufficient activity							
Yes	196 (48.2)	0 (0)	196 (100)	—	—	—	—
No	211 (51.8)	211 (100)	0 (0)				
Sedentary behaviors							
Yes	184 (45.2)	74 (40.2)	110 (59.8)	2.368 (1.588–3.531)	<0.001	2.688 (1.756–4.112)	<0.001
No	223 (54.8)	137 (61.4)	86 (38.6)	Ref.		Ref.	

Note: COR: Crude Odds Ratios; AOR: Adjusted Odds Ratio.

**Table 2:** Regression analysis predicting sedentary behaviors.

Variables	No	Yes	Bivariate Analysis		Multivariable Analysis	
	n (%)	n (%)	COR (95% CI)	*p*-Value	AOR (95% CI)	*p*-Value
Gender						
Female	118 (52.9)	105 (47.1)	1.183 (0.798–1.752)	0.403	—	—
Male	105 (57.1)	79 (42.9)	Ref.			
Age						
≤20 years	44 (53.7)	38 (46.3)	1.152 (0.581–2.283)	0.686	—	—
21–23 years	147 (54.6)	122 (45.4)	1.107 (0.619–1.979)	0.733	—	—
≥24 years	32 (57.1)	24 (42.9)	Ref.			
Medical university						
Public	133 (51)	128 (49)	1.547 (1.024–2.337)	0.038	1.319 (0.834–2.085)	0.237
Private	90 (61.6)	56 (38.4)	Ref.		Ref.	
Year of study						
2nd	30 (56.6)	23 (43.4)	0.767 (0.291–2.017)	0.59	0.868 (0.305–2.474)	0.792
3rd	57 (54.3)	48 (45.7)	0.842 (0.347–2.046)	0.704	1.017 (0.387–2.669)	0.973
4th	70 (64.2)	39 (35.8)	0.557 (0.229–1.358)	0.198	0.65 (0.244–1.729)	0.388
5th (Final)	54 (46.6)	62 (53.4)	1.148 (0.477–2.766)	0.758	1.425 (0.547–3.713)	0.469
1st	12 (50)	12 (50)	Ref.		Ref.	
Residence						
Rural	7 (38.9)	11 (61.1)	2.034 (0.651–6.356)	0.222	2 (0.603–6.638)	0.257
Urban	194 (55.4)	156 (44.6)	1.041 (0.534–2.028)	0.907	1.182 (0.578–2.415)	0.647
Semi-urban	22 (56.4)	17 (43.6)	Ref.		Ref.	
Family type						
Nuclear	172 (55.3)	139 (44.7)	Ref.			
Joint	51 (53.1)	45 (46.9)	1.092 (0.69–1.728)	0.708	—	—
BMI						
Normal	139 (51.7)	130 (48.3)	1.683 (0.927–3.057)	0.087	2.181 (1.135–4.193)	0.019
Overweight/obese	48 (58.5)	34 (41.5)	1.275 (0.632–2.571)	0.497	1.359 (0.632–2.923)	0.432
Underweight	36 (64.3)	20 (35.7)	Ref.		Ref.	
Cigarette smoking						
Yes	5 (45.5)	6 (54.5)	1.47 (0.441–4.895)	0.531	—	—
No	218 (55.1)	178 (44.9)	Ref.			
Do you have any COVID-19 related symptoms?						
Yes	8 (32)	17 (68)	3.32 (1.163–9.477)	0.025	2.888 (0.919–9.074)	0.069
No	190 (55.7)	151 (44.3)	1.242 (0.64–2.41)	0.522	1.324 (0.658–2.667)	0.432
May be	25 (61)	16 (39)	Ref.		Ref.	
Have you been diagnosed with COVID-19 by a health professional?						
Yes	2 (28.6)	5 (71.4)	3.087 (0.592–16.098)	0.181	1.66 (0.256–10.753)	0.595
No	221 (55.3)	179 (44.8)	Ref.		Ref.	
Have you been living with a person diagnosed with COVID-19?						
Yes	6 (33.3)	12 (66.7)	2.523 (0.928–6.86)	0.070	1.832 (0.597–5.617)	0.290
No	217 (55.8)	172 (44.2)	Ref.		Ref.	
Are you following COVID-19 Standard Operating Procedures (SOPs)?						
Yes	181 (53.7)	156 (46.3)	1.293 (0.724–2.31)	0.386	—	—
No	9 (60)	6 (40)	1 (0.312–3.207)	1.000	—	—
Not sure	33 (60)	22 (40)	Ref.			
Insufficient activity						
Yes	86 (43.9)	110 (56.1)	2.368 (1.588–3.531)	<0.001	2.53 (1.661–3.853)	<0.001
No	137 (64.9)	74 (35.1)	Ref.			

Note: COR: Crude Odds Ratios; AOR: Adjusted Odds Ratio.

## Data Availability

The data presented in this study are available on request from the corresponding author.
